# The senescence-inducing factor IGFBP7 and risk of atrial fibrillation: findings from the PREVEND study

**DOI:** 10.1038/s44325-025-00079-1

**Published:** 2025-08-12

**Authors:** Navin Suthahar, Ron T. Gansevoort, Thomas F. Kok, Maissa El-Qendouci, Teun B. Petersen, Stephan J. L. Bakker, Eric Boersma, Kevin Damman, Isabella Kardys, Michiel Rienstra, Rudolf A. de Boer

**Affiliations:** 1 Department of Cardiology, Cardiovascular Institute, Thorax Center, Erasmus MC, Rotterdam, The Netherlands; 2 Division of Nephrology, Department of Internal Medicine, University Medical Center Groningen, University of Groningen, Groningen, The Netherlands; 3 Department of Cardiology, University Medical Center Groningen, University of Groningen, Groningen, The Netherlands

**Keywords:** Atrial fibrillation, Atrial fibrillation

## Abstract

Experimental evidence supports the role of cellular senescence in the pathophysiology of atrial fibrillation (AF) and suggests that Insulin-like Growth Factor Binding Protein-7 (IGFBP7) is an important senescence-inducing factor. Whether elevated IGFBP7 levels precede the development of AF remains unknown. We measured IGFBP7 levels in plasma of 5884 adult participants without prevalent AF (mean age 53.6 ± 12.1 years; 51.5% women) from the PREVEND community-based cohort (2001–2004). Incident AF was ascertained from hospital and study electrocardiograms. During a median follow-up of 6.4 (5.9–6.9) years, 154 participants (2.6%) developed AF. In Cox proportional hazards models, IGFBP7 was associated with increased risk for incident AF (unadjusted hazard ratio [HR]: 1.85 per 1-SD increase in log-IGFBP7; 95% confidence interval [CI]: 1.65–2.07) which remained significant after adjustment for clinical variables (HR: 1.28; 95% CI: 1.07–1.52). Based on these data, we conclude that IGFBP7, a senescence-inducing factor, is associated with the risk of developing AF in community-dwelling adults.

## Introduction

Atrial fibrillation (AF) is the most common cardiac arrhythmia and a major contributor to cardiovascular morbidity and mortality^1^. Key risk factors for AF include age, genetics, lifestyle, and pre-existing heart disease, with age being the most significant^2^. As the global population continues to age^3^, the prevalence and incidence of AF are also expected to increase sharply^2^. This is because AF disproportionately affects the elderly, with its incidence rising steeply from ~0.9 per 1000 person-years at ages 40–49 years to over 17 per 1000 person-years in those over 70 years^4^.

Cellular senescence plays a crucial role in the development of AF by accelerating cardiovascular ageing and promoting myocardial fibrosis^5^. These changes cause adverse myocardial and atrial remodeling, which can directly influence structural as well as electrical properties of the cardiac tissue^6,7^—contributing to the development of AF. However, clinical studies exploring the link between senescence and AF remain limited, which may, in part, be due to the lack of reliable circulating markers reflecting senescence.

Insulin-like growth factor binding protein-7 (IGFBP7) is a senescence-related marker that can be reliably measured in circulation^8,9^. Recent studies suggest that IGFBP7 contributes to cardiovascular senescence by stimulating IGF-1 receptor-dependent suppression of Forkhead Box O3a (FOXO3a)^10^, a transcription factor involved in DNA repair and oxidative stress defense^11–13^. Whereas increased circulating IGFBP7 levels are known to be associated with heart failure (HF) development and progression^14–16^, large population-based studies examining its relationship with AF are lacking.

Given the role of cellular senescence in AF pathogenesis, we hypothesized that higher circulating IGFB7 levels, reflecting increased cellular senescence, would be associated with a greater risk of developing AF. Therefore, the objective of our study was to measure circulating IGFBP7 in community-dwelling adults and to examine its association with incident AF.

## Results

### Plasma IGFBP7 levels

The study included 5884 individuals who were free of AF at baseline. The mean (SD) age of the overall population was 53.6 (12.1) years, and 51.5% were females. The distribution of IGFBP7 in the PREVEND general population is shown in Supplementary Fig. 1 and Supplementary Table 1. Median IGFBP7 level in the total population was 84.7 µg/L, and the range varied from 14.0 to 320.4 µg/L. The distribution of IGFBP7 was broadly similar in both sexes, but median IGFBP7 levels were significantly higher in males than in females (89.1 vs 79.7 µg/L, *P* < 0.001).

### Participant characteristics

We divided the study population according to incident AF status, and results are presented in Table 1. Compared to participants not developing AF, those developing AF had a higher age (53.3 ± 12.0 vs 65.4 ± 8.4 years, *P* < 0.001), were less often females (52.1% vs 27.9%, *P* < 0.001), and had higher levels of IGFBP7 (84.3 [74.8, 95.4] vs 98.1 [97.3, 111.0] µg/L, *P* < 0.001).

**Table 1:** Participant characteristics according to incident atrial fibrillation status

Characteristics	No incident AF	Incident AF	*P* value
	*N* = 5730	*N* = 154	
IGFBP7 (median, P25–P75), µg/L	84.3 (74.8–95.4)	98.1 (87.3–111.0)	<0.001
Age (mean, SD), years	53.3 (12.0)	65.4 (8.4)	<0.001
Female sex, *n* (%)	2988 (52.1)	43 (27.9)	<0.001
Height (mean, SD), cm	172.5 (9.5)	175.2 (9.4)	<0.001
Weight (mean, SD), kg	79.2 (14.3)	87.9 (14.6)	<0.001
Relative fat mass, %	32.0 (7.3)	32.4 (6.9)	0.52
Body mass index, kg/m^2^	26.6 (4.3)	28.6 (4.4)	<0.001
SBP (mean, SD), mm Hg	125.7 (18.7)	136.7 (21.6)	<0.001
Antihypertensive medication, *n* (%)	1208 (21.1)	89 (57.8)	<0.001
Smoking			<0.001
Current, *n* (%)	1563 (27.3)	39 (25.3)	
Past, *n* (%)	2401 (41.9)	87 (56.5)	
Diabetes, *n* (%)	359 (6.3)	22 (14.4)	<0.001
Myocardial infarction or stroke, *n* (%)	384 (6.7)	45 (29.2)	<0.001
Heart failure, *n* (%)	36 (0.6)	14 (9.1)	<0.001
NT-proBNP (median, P25–P75), ng/L	41.0 (21.0–78.0)	153.5 (70.0–304.0)	<0.001
UAE (median, P25–P75), mg/24 h	8.1 (5.9–13.5)	12.2 (7.5–37.7)	<0.001
eGFR (mean, SD), mL/min/1.73 m^2^	92.5 (16.4)	79.4 (18.2)	<0.001

Continuous variables are presented as mean (standard deviation) or as a median (percentile 25 to percentile 75), and categorical variables as *n* (%).

*AF* atrial fibrillation, *IGFBP7* insulin-like growth factor binding protein-7, *SBP* systolic blood pressure, *NT-proBNP* N-terminal pro-B-type natriuretic peptide, *UAE* 24-h urinary albumin excretion, *eGFR* estimated glomerular filtration rate.

### Associations of plasma IGFBP7 with incident AF

During a median (P25–P75) follow-up of 6.4 (5.9–6.9) years, 154 participants (2.6%) developed AF, corresponding to an incidence rate of 4.2 new AF events per 1000 person-years.

**Fig. 1:**
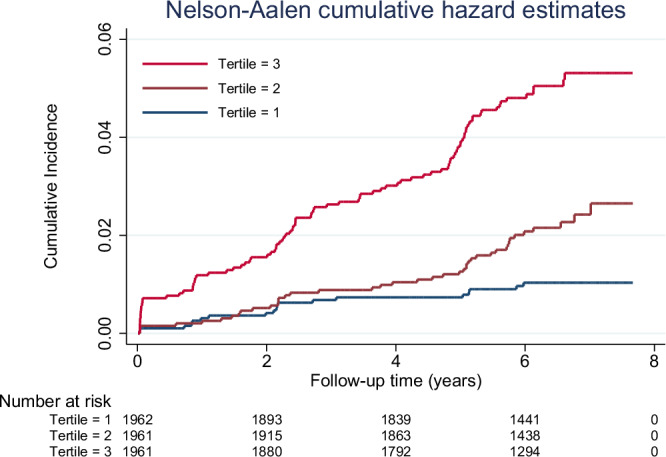
Cumulative incidence of atrial fibrillation across sex-pooled IGFBP7 tertiles. This figure illustrates the cumulative incidence of atrial fibrillation (AF) across tertiles of insulin-like growth factor binding protein-7 (IGFBP7). The *y*-axis represents the cumulative incidence of AF, while the *x*-axis shows the time from study inclusion to AF event or censoring. The cumulative incidence curves are color-coded: tertile 1 is shown in black, tertile 2 in cranberry, and tertile 3 in red. The table below (number at risk) indicates the number of participants at risk at different time points for each IGFBP7 tertile.

Population characteristics according to sex-pooled IGFBP7 tertiles are presented in Table 2. The cumulative incidence were significantly different across the sex-pooled IGFBP7 tertiles (*P* < 0.001), with participants with higher IGFBP7 levels having the highest cumulative incidence of AF (Fig. 1). Incidence rates per 1000 person-years of AF also increased progressively across IGFBP7 tertiles with 1.6 events in the first tertile; 3.4 in the second tertile; and 7.9 in the third tertile.

**Table 2:** Participant characteristics according to sex-pooled IGFBP7 tertiles

Characteristics	Tertile 1	Tertile 2	Tertile 3	*P* value
	*n* = 1962	*n* = 1961	*n* = 1961	
IGFBP7 (median, P25–P75), µg/L	70.8 (64.5–77.1)	84.7 (79.5–88.9)	101.2 (95.7–110.1)	<0.001
Age (mean, SD), years	47.1 (9.4)	52.4 (10.6)	61.2 (11.6)	<0.001
Female sex, *n* (%)	1011 (51.5)	1010 (51.5)	10101 (51.5)	1.00
Height (mean, SD), cm	173.2 (9.5)	173.0 (9.5)	171.7 (9.5)	<0.001
Weight (mean, SD), kg	78.6 (13.9)	79.4 (14.3)	80.4 (14.7)	<0.001
Relative fat mass, %	31.1 (6.9)	31.8 (7.2)	33.2 (7.5)	<0.001
Body mass index, kg/m^2^	26.2 (4.0)	26.5 (4.2)	27.3 (4.6)	<0.001
SBP (mean, SD), mm Hg	120.6 (14.7)	124.4 (18.0)	132.9 (21.1)	<0.001
Antihypertensive medication, *n* (%)	236 (12.1)	367 (18.7)	694 (35.4)	<0.001
Smoking				<0.001
Current, *n* (%)	626 (31.9)	549 (28.0)	427 (21.8)	
Past, *n* (%)	733 (37.4)	814 (41.5)	941 (48.0)	
Diabetes, *n* (%)	74 (3.8)	101 (5.2)	206 (10.6)	<0.001
MI or stroke, *n* (%)	87 (4.4)	129 (6.6)	213 (10.9)	<0.001
Heart failure, *n* (%)	5 (0.3)	11 (0.6)	34 (1.7)	<0.001
NT-proBNP (median, P25–P75), ng/L	35.0 (18.0–61.0)	38.0 (21.0–70.0)	60.0 (30.0–119.0)	<0.001
UAE (median, P25–P75), mg/24 h	7.6 (5.8–11.1)	8.0 (5.9–12.4)	9.7 (6.3–20.2)	<0.001
eGFR (mean, SD), mL/min/1.73 m^2^	101.3 (12.5)	94.5 (13.2)	80.6 (16.7)	<0.001

Continuous variables are presented as mean (standard deviation) or as a median (percentile 25 to percentile 75), and categorical variables as *n* (%).

*AF* atrial fibrillation, *IGFBP7* insulin-like growth factor binding protein-7, *SBP* systolic blood pressure, *NT-proBNP* N-terminal pro-B-type natriuretic peptide, *UAE* 24-h urinary albumin excretion, *eGFR* estimated glomerular filtration rate.

**Fig. 2:**
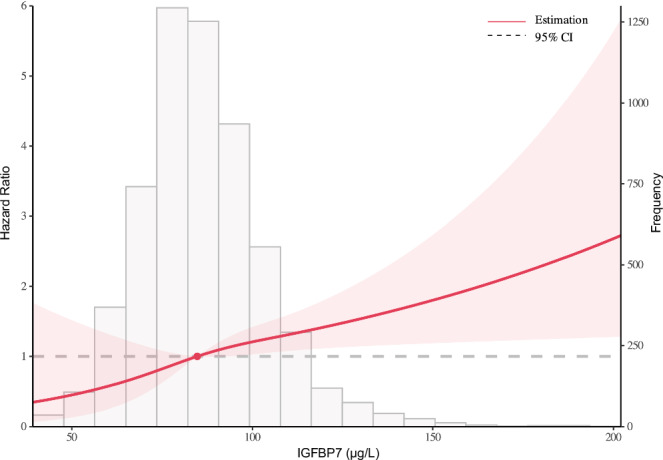
Restricted cubic spline curve depicting the association between IGFBP7 levels and incident atrial fibrillation. This figure illustrates the association between insulin-like growth factor binding protein-7 (IGFBP7) levels and the hazard of incident atrial fibrillation (AF) using a restricted cubic spline model. The *x*-axis represents IGFBP7 concentrations (µg/L), and the *y*-axis represents the hazard ratio for AF. The solid red line denotes the hazard ratio, with light-red shading indicating the 95% confidence intervals. Knots were placed at the 5th, 35th, 65th, and 95th percentiles of IGFBP7. The model was adjusted for age, sex, weight, height, systolic blood pressure, antihypertensive medication, smoking, type 2 diabetes, history of myocardial infarction or stroke, history of heart failure, and estimated glomerular filtration rate. The gray-shaded background (histogram) represents the distribution of IGFBP7 in the study population.

In unadjusted Cox regression models, IGFBP7 levels were significantly associated with increased risk for incident AF (hazard ratio (HR): 1.85 per 1-SD increase in log IGFBP7; 95% confidence interval (CI): 1.65–2.07). This association remained statistically significant after adjustment for age and sex (HR: 1.34; 95% CI: 1.15–1.56), and after further adjustment for weight, height, systolic blood pressure (BP), antihypertensive medication, smoking, type 2 diabetes, history of myocardial infarction or stroke, history of HF and estimated glomerular filtration rate (eGFR) (HR: 1.28; 95% CI: 1.07–1.52) (Table 3, Fig. 2). Results did not materially change when we additionally adjusted for NT-proBNP (HR: 1.25; 95% CI: 1.04–1.50), interim HF (HR: 1.28; 95% CI: 1.07–1.53) or competing risk of death (HR: 1.26; 95% CI: 1.05–1.53).

**Table 3:** Association of IGFBP7 levels with incident atrial fibrillation

	Hazard ratio (95% CI)	*P* value
Model 1	1.85 (1.65, 2.07)	<0.001
Model 2	1.34 (1.15, 1.56)	<0.001
Model 3	1.26 (1.07, 1.48)	0.005
Model 4	1.28 (1.07, 1.52)	0.007

Model 1 was unadjusted; model 2 was adjusted for age and sex; model 3 was additionally adjusted for weight, height, systolic blood pressure, antihypertensive medication, smoking, history of type 2 diabetes, history of myocardial infarction or stroke, and history of heart failure. IGFBP7 was log-transformed and standardized separately for males and females.

For clinical relatability, we also provided HRs per 20 µg/L increase in IGFP7 levels, which approximately corresponds to a 1-SD increase of IGFBP7 (18.3 µg/L) in the PREVEND population (Supplementary Table 2). Finally, in post-hoc analysis, we found no significant interaction between IGFBP7 levels and age, sex, obesity, prevalent HF, prevalent cardiovascular disease (CVD), renal dysfunction, or microalbuminuria—with regard to AF incidence (Supplementary Table 3).

## Discussion

In the current study, including 5884 community-dwelling adults free of prevalent AF, we report for the first time that IGFBP7, a senescence-inducing factor, is associated with *incident* AF. Our findings expand on observations from smaller studies examining cross-sectional associations of IGFBP7 and AF. Specifically, IGFBP7 levels were associated with *prevalent* AF in an elderly community-based cohort^17^ and also with *prevalent* AF in HF patients with reduced ejection fraction^18^. Additionally, in a cohort of patients diagnosed with AF, higher IGFBP7 levels were associated with future HF-related hospitalization^19^.

These observations suggest that IGFBP7 may have some predictive value in the development and progression of AF. However, considering that IGFBP7 has a ubiquitous expression (Supplementary Fig. 2), and only a fraction of the circulating IGFBP7 pool might arise from the cardiac tissue^20^, its potential to predict/diagnose cardiac-specific disorders such as AF, when used as a single marker, may be limited. In this regard, a recent study examining the value of biomarker clusters found that a combination of cardiac-specific proteins (NT-proBNP and bone morphogenetic protein (BMP)-10) along with non-cardiac proteins (IGFBP7, angiopoietin 2, and growth differentiation factor-15) effectively identified high risk in AF patients^21^.

Besides its suggestive value in AF risk estimation, IGFBP7 should also be considered from a pathophysiologic perspective—as a cardiovascular geroprotein amenable to therapy. Indeed, there *is* experimental evidence showing that cardiovascular ageing/senescence can be modified by inhibiting IGFBP7. Specifically, in a pressure-overload mouse model, IGFBP7 accelerated HF progression by promoting cardiac senescence, whereas antibody-mediated IGFBP7 neutralization (in vivo) attenuated HF progression^10^. Additionally, in a similar mouse model of HF, IGFBP7 produced by senescent endothelial cells promoted cardiac dysfunction, and a vaccine targeting IGFB7 ameliorated cardiac dysfunction^22^.

Cardiovascular senescence and fibrosis are involved not only in the pathogenesis of HF^23,24^, but also in the pathogenesis of AF^5–7^. As higher IGFBP7 levels, reflecting increased senescence, precede the development of AF in community-dwelling adults, we hypothesize that IGFBP7 inhibition may also emerge as a potential treatment for AF. To this end, validation of our results in other population-based cohorts (including AF patients), along with carefully designed mechanistic studies investigating the role of IGFBP7 inhibition in experimental AF, is first needed.

This is one of the first studies investigating the association of IGFBP7 with *incident* AF in community-dwelling adults. Other strengths include a detailed clinical assessment, an almost 1:1 sex ratio, and adjudicated AF outcomes^25^. Notwithstanding, we acknowledge the following limitations. First, due to the observational design of our study, residual confounding cannot be ruled out, and causal relations between IGFBP7 and AF development cannot be established. Second, the PREVEND study by design enrolled a higher proportion of individuals with mildly elevated urinary albumin excretion (UAE); however, this is unlikely to affect the interpretation, as previous research has demonstrated that findings from the PREVEND cohort align well with those from broader population cohorts like the Framingham Heart Study^26^. Third, we acknowledge that the PREVEND cohort was established several years ago, and changes in population demographics and risk profiles over time could potentially impact the external validity of our results. Finally, this study was conducted in a predominantly White population, which may limit the generalizability of our findings to other ethnicities/population groups. Therefore, our results should be validated in more recent cohorts with contemporary follow-up and in different ethnicities/population groups.

In conclusion, we report that IGFBP7, a senescence-inducing factor, is associated with increased risk for incident AF among community-dwelling adults. Future studies should explore IGFBP7 inhibition as a potential treatment for AF.

## Methods

### Study population

The Prevention of Renal and Vascular End-stage Disease (PREVEND) study was founded in 1997 as a prospective community-based study^25,27^. In brief, all inhabitants (between 28 and 75 years) of the city of Groningen, the Netherlands, were invited (*n* = 85,421), and 47.8% (*n* = 40,856) responded. Individuals with UAE > 10 mg/L (*n* = 7768) in their morning urine, as well as a randomly selected control group with UAE < 10 mg/L (*n* = 3394), were selected to attend the PREVEND outpatient clinic^28^. After excluding participants with insulin-dependent diabetes, pregnant women, and individuals unable/unwilling to participate, a final total of 8592 individuals completed the (first) PREVEND screening program (1997–1998)^28^, during which multiple demographic, anthropometric, and health-related factors were assessed. Fasting venous blood samples and two 24-h urine samples per person were collected and stored at −80 °C. Participants were then regularly screened at ~3-year intervals at the PREVEND outpatient clinic. During each follow-up visit, a similar screening protocol was followed, including the collection and storage of fasting venous blood samples and two 24-h urine samples per person; additionally, a 12-lead ECG was also taken. IGFBP7 was measured in samples from the second visit, which was attended by 6894 participants. Therefore, the second PREVEND visit was considered as the *baseline* for the present analyses.

From the 6894 participants attending the baseline visit (2001–2004), we excluded those with prevalent AF (*n* = 85), unknown rhythm status (*n* = 178), and unavailable plasma samples/IGFBP7 measurements (*n* = 733). As IGFBP7 displayed a strong correlation with renal function^14^, we additionally excluded individuals with eGFR less than 30 mL/min/1.73 m^2^ (*n* = 14), resulting in 5884 participants for analyses. Participant characteristics of the baseline sample (*n* = 6894) and the sample used for the current analyses (*n* = 5884) are shown in Supplementary Table 4. Ethical approval was obtained from the local medical ethics committee of the University Medical Center Groningen (MEC96/01/022); all participants signed informed consent, and the study was conducted in accordance with the Declaration of Helsinki.

### Baseline assessment

All anthropometric measurements were performed in a standing position. Waist circumference (WC) was measured midway between the lowest rib and the iliac crest at the end of expiration. Relative fat mass was calculated using height and WC with the following equation: 64−(20 × height/WC) + (12 × sex), with sex = 0 (men) and sex = 1 (women)^28^. Body mass index was calculated as weight/height^2^ (kg/m^2^). BP was calculated as the average of two seated measurements. Diabetes was defined as fasting glucose 126 mg/dL (7.0 mmol/L) or higher, a non-fasting glucose of 200 mg/dL (11.1 mmol/L) or higher, or hypoglycaemic medication usage. Prevalent CVD, defined as a history of myocardial infarction or stroke, was obtained from a structured questionnaire, which included criteria such as hospitalization lasting 3 days or more due to the specified condition. This data collection was supplemented by an examination of medical records. The history of HF was obtained from hospital charts. Smoking behavior was self-reported and was defined as current smoking (smoking at present or smoking cessation within the previous year) or past smoking (smoking cessation over 1 year), or never smoking. Plasma glucose was measured by dry chemistry (Eastman Kodak, Rochester, NY, USA). Plasma N-terminal pro-B-type natriuretic peptide (NT-proBNP) was measured using an electro-chemiluminescence sandwich immunoassay (Elecsys proBNP, Roche Diagnostics, Mannheim, Germany). Urinary albumin concentration was determined by nephelometry (BNII, Dade Behring Diagnostic, Marburg, Germany)^27^. eGFR was calculated using the Chronic Kidney Disease Epidemiology Collaboration (CKD-EPI) combined creatinine-cystatin C equation.

### IGFBP7 measurements

IGFBP7 was measured in plasma samples using a high-precision precommercial COBAS Elecsys assay (Roche Diagnostics GmbH, Penzberg, Germany)^14^. The detection method for IGFBP7 was a sandwich immunoassay developed on the Elecsys® platform utilizing electro-chemiluminescence detection by Roche Diagnostics GmbH (Mannheim, Germany). Mouse monoclonal antibodies were produced and tested to specifically identify IGFBP7. The precision within-run coefficient of variation for IGFBP7 was 2%, and the limit of detection was 0.01 µg/L.

### Incidence of atrial fibrillation

Ascertainment of incident AF has been described in detail previously^25,29,30^. In brief, incident AF was diagnosed if AF was present on a 12-lead ECG obtained during scheduled PREVEND visits (ie, visit 3 or 4), or during an outpatient visit or hospital admission in either of the two hospitals of the city of Groningen. All ECGs were digitally stored and were first screened electronically for atrial flutter, ectopic atrial rhythm, or the absence of PR interval. Then, two independent observers (ie, physicians with experience in evaluating ECGs) reviewed all suspected AF cases as determined by electronic screening. When there was an inconsistency between the observers or when both observers agreed on the diagnosis of atrial flutter or AF, the ECGs were validated by 2 independent cardiologists. For the date of the incident AF, the date of the first ECG, with a definite diagnosis of AF, was used^30^. The follow-up duration was calculated as the time between the baseline (ie, visit 2) and incident AF, death, or 31 December 2008.

### Statistical analyses

Continuous variables are presented as means (standard distributions) for normally distributed data or as medians (25th–75th percentiles) for skewed data. Categorical variables are presented as counts (percentages). Differences in continuous variables between groups were assessed using Student’s *t*-test or the Mann–Whitney *U* test for two groups, and ANOVA or the Kruskal–Wallis test for more than two groups. Categorical variables were compared using chi-square tests and Fisher’s exact tests.

We first examined the association between IGFBP7 and incident AF using Cox proportional hazards regression (after checking the proportional hazard assumption) using four models. The first model was unadjusted; the second model was adjusted for age and sex; the third model was additionally adjusted for components of CHARGE-AF model (weight, height, systolic BP, antihypertensive medication, smoking, type 2 diabetes, history of myocardial infarction or stroke, history of HF)^31^; and the fourth model was additionally adjusted for eGFR. For these analyses, IGFBP7 was log-transformed and standardized separately for males and females.

Additionally, we examined associations of IGFBP7 and incident AF per 20 µg/L increase in IGFBP7 levels. In post-hoc subgroup analyses, we explored age-adjusted and/or sex-adjusted associations of IGFBP7 with incident AF according to age, sex, obesity, prevalent HF, prevalent CVD, renal dysfunction (eGFR < 60 mL/min/1.63 m^2^), and microalbuminuria (UAE ≥ 30 mg/24 h).

We report our findings as (HRs) and their corresponding 95% CIs. All analyses were performed using STATA version 14.0 (Stata Corp., College Station, TX, USA). All tests were two-tailed, and *p* values < 0.05 were considered statistically significant.

## Supplementary information

Supplementary File

## Data Availability

The dataset analyzed during the current study is available in the PREVEND repository https://umcgresearch.org/w/prevend and can be obtained after an application is approved by the PREVEND board.

## Code availability

Codes of Stata version 14 (Stata Corp., College Station, TX, USA) for statistical analysis of this study are available upon request from Navin Suthahar.
