# Supplementary material for: The senescence-inducing factor IGFBP7 and risk of atrial fibrillation: findings from the PREVEND study
Source: NPJ Cardiovasc Health. 2025 Aug 12;2:42. doi: 10.1038/s44325-025-00079-1 (PMC12343286; doi:10.1038/s44325-025-00079-1)
Supplement: Supplementary file 1 — Supplementary File [file 44325_2025_79_MOESM1_ESM.pdf]

Supplementary Figure 1. Distribution of IGFP7 in the PREVEND general population cohort

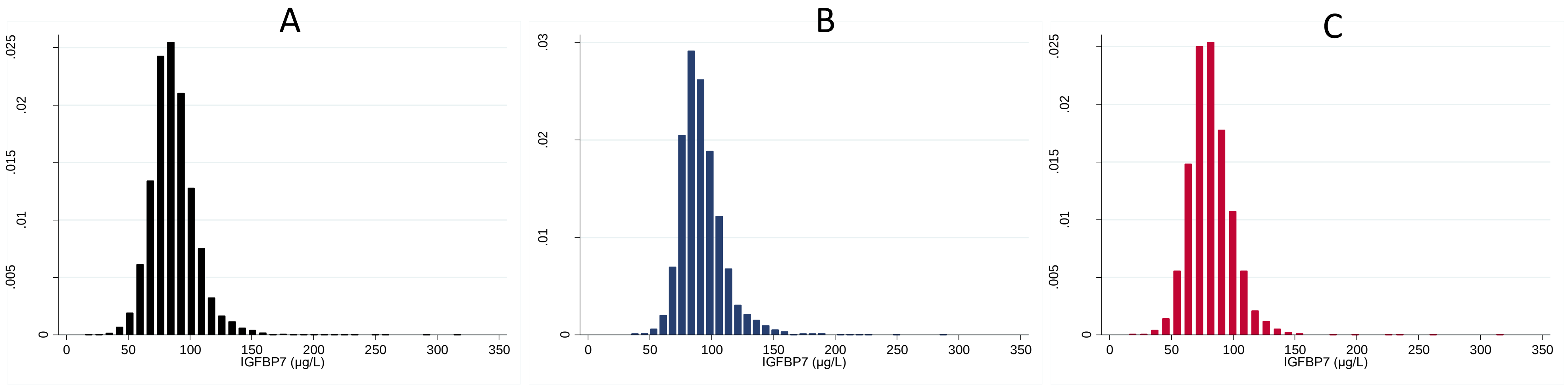

This figure illustrates the distribution of insulin-like growth factor binding protein 7 (IGFBP7) levels (µg/L) in the PREVEND cohort. **Panel A** shows the overall population (black), **Panel B** shows represents males (blue), and **Panel C** represents females (cranberry).

Supplementary Figure 2. Tissue-Specific RNA Expression of IGFBP7

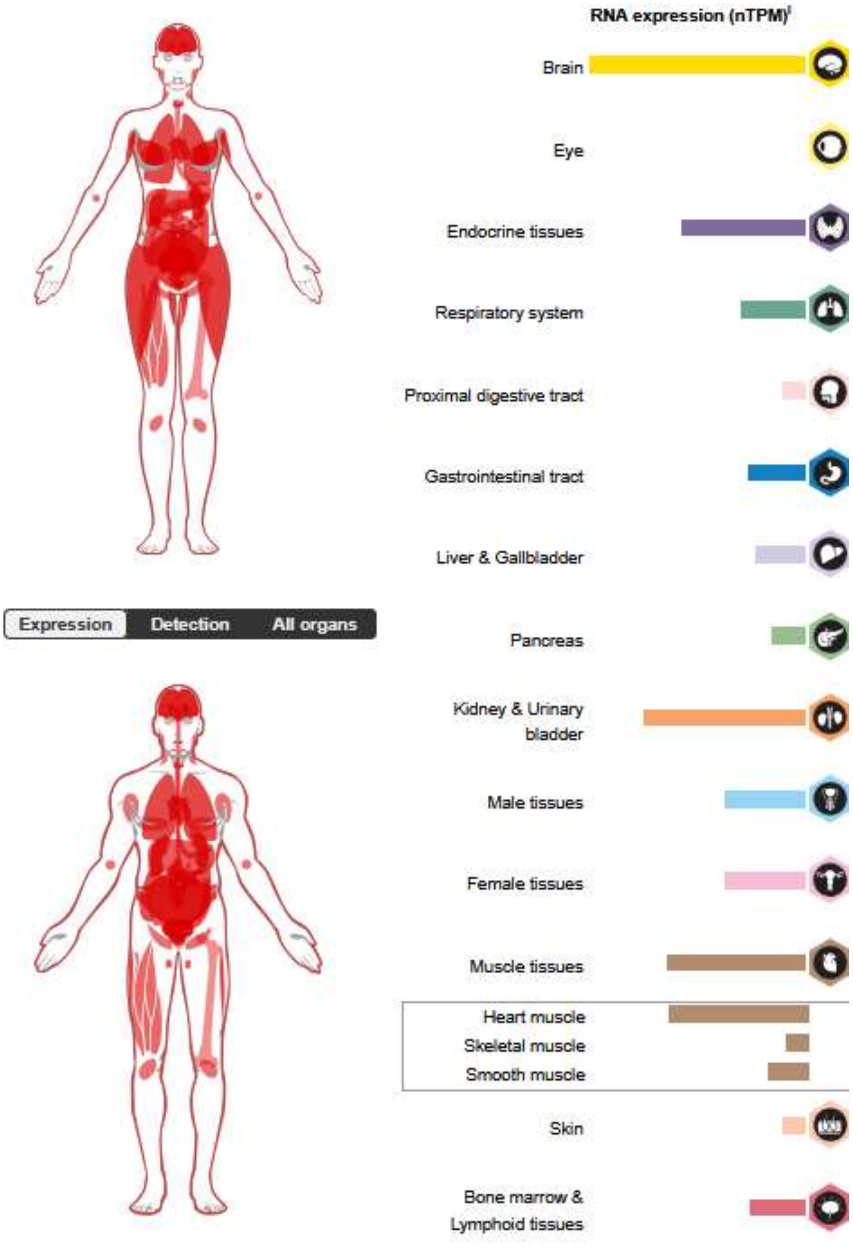

IGFBP7 has a ubiquitous expression profile with the highest expression in the brain, kidney & urinary bladder, and cardiac tissue. Reference: https://www.proteinatlas.org/ENSG00000163453-IGFBP7/tissue

Supplementary Table 1. Percentiles of IGFBP7 in the PREVEND cohort

Percentiles	Total<br>n = 5884	Males<br>n = 2853	Females<br>N = 3031
1	50.4	59.6	45.3
5	60.8	69.2	57.0
10	65.9	73.9	62.0
25	75.1	80.4	70.3
**50**	**84.7**	**89.1**	**79.7**
75	95.7	99.8	91.0
90	107.7	111.9	102.7
95	116.3	122.5	110.2
99	142.8	149.6	130.7

IGFBP7 levels ranged from 14.0 to 320.4 μg/L in the total population, 34.3 to 291.3 μg/L in males, and 14.0 to 320.4 μg/L in females. The mean (SD) of IGFBP7 was 86.5 (18.8) μg/L in the total population; 91.8 (18.2) μg/L in males, and 81.5 (18.0) μg/L in females.

Supplementary Table 2. Associations of IGFBP7 with incident Atrial Fibrillation

	**Hazard Ratio (95% CI)**	**P-value**
Model 1	1.53 (1.43, 1.63)	<0.001
Model 2	1.26 (1.13, 1.40)	<0.001
Model 3	1.19 (1.07, 1,34)	0.002
Model 4	1.20 (1.06, 1.35)	0.004

Model 1 was unadjusted; model 2 was adjusted for age and sex; model 3 was additionally adjusted for weight, height, systolic blood pressure, antihypertensive medication, smoking, history of type-2 diabetes, history of myocardial infarction or stroke and history of prevalent heart failure. Hazard ratios are per 20 μg/L increase in IGFBP7 levels.

Supplementary Table 3. Exploratory subgroup / interaction analyses

	No	Yes	
	HR (95% CI)	HR (95% CI)	P-interaction
Older age*	1.23 (1.05, 1.45)	1.33 (1.16, 1.53)	0.778
Female sex	1.25 (1.09, 1.45)	1.26 (1.08, 1.48)	0.812
Obesity (BMI)*	1.22 (1.04, 1.43)	1.31 (1.13, 1.51)	0.951
Obesity (RFM)*	1.17 (0.98, 1.39)	1.34 (1.16, 1.54)	0.609
Prevalent HF	1.22 (1.08, 1.38)	1.97 (1.27, 3.06)	0.154
Prevalent CVD*	1.16 (0.97, 1.39)	1.35 (1.16, 1.56)	0.715
Prevalent CKD*	1.30 (0.99, 1.71)	1.21 (1.06, 1.39)	0.884
Microalbuminuria*	1.23 (1.07 1.42)	1.22 (1.02, 1.47)	0.586

The stratified models were adjusted for age and sex. While examining associations of sex with incident AF, models were adjusted only for age. Hazard ratios are given per 20 µg/L increase in IGFBP7 levels.
Abbreviations: BMI, body mass index; RFM, reletive fat mass; HF, heart failure; CVD, cardiovascular disease; CKD, chronic kidney; NT-proBNP, N-terminal pro-B-type natriuretic peptide.
* Older age was defined as age ≥ 70 years; BMI based obesity was defined as BMI ≥ 30 kg/m^2; RFM based obesity was defined as RFM ≥ 30 in men & RFM ≥ 40 in women; CKD was defined as estimated glomerular filtration rate ≤ 60 mL/min/1.63m^2; CVD was defined as myocardial infarction or stroke; Microalbuminuria was defined as urinary albumin excretion ≥ 30 mg per 24 hours.

Supplementary Table 4. PREVEND participant characteristics: total sample vs study sample

Characteristics	Total Sample	Study Sample
	**N = 6894**	**N = 5884**
IGFBP7 (median, P25-P75), μg/L	84.8 (75.1-95.9)	84.7 (75.1-95.7)
Age (mean, SD), years	53.9 (12.1)	53.6 (12.0)
Female sex, n (%)	3450 (50.0)	3031 (51.5)
Height (mean, SD), cm	172.7 (9.5)	172.6 (9.5)
Weight (mean, SD), kg	79.9 (14.6)	79.5 (14.3)
Relative fat mass, %	31.9 (7.3)	32.0 (7.3)
Body mass index, kg/m^2	26.8 (4.4)	26.7 (4.3)
SBP (mean, SD), mm Hg	126.5 (18.9)	126.0 (18.8)
Antihypertensive medication, n (%)	1539 (22.4)	1297 (22.1)
Current smokers, n (%)		
Current, n (%)	1917 (27.8)	1602 (27.2)
Past, n (%)	2925 (42.4)	2488 (42.3)
Diabetes, n (%)	483 (7.1%)	381 (6.5%)
Myocardial infarction or stroke, n (%)	505 (7.3)	429 (7.3)
Heart failure, n (%)	64 (0.9)	50 (0.8)
NT-proBNP (median, P25-P75), ng/L	42.0 (21.0-82.0)	42.0 (22.0-81.0)
UAE (median, P25-P75), mg/24h	8.8 (6.1-16.5)	8.2 (6.0-13.7)
eGFR (mean, SD), mL/min/1.73m^2	91.8 (17.3)	92.2 (16.6)

Continuous variables are presented as mean (standard deviation) or as a median (percentile 25 – percentile 75), and categorical variables as n (%). Abbreviations: SBP, systolic blood pressure; IGFBP7, insulin like growth factor binding protein-7; eGFR, estimated glomerular filtration rate; NT-proBNP, N-terminal pro-B-type natriuretic peptide; UAE, 24-hour urinary albumin excretion.
